# Pain Management Interventions in Lumbar Spinal Stenosis: A Literature Review

**DOI:** 10.7759/cureus.44116

**Published:** 2023-08-25

**Authors:** Kashif N Malik, Curren Giberson, Matthew Ballard, Nathan Camp, Justin Chan

**Affiliations:** 1 Physical Medicine and Rehabilitation, Casa Colina Hospital, Pomona, USA; 2 Physical Medicine and Rehabilitation, Western University of Health Sciences, Pomona, USA

**Keywords:** minimally invasive lumbar decompression, physical medicine and rehabilitation, epidural steroid injection, interventional pain medicine, vertiflex, lumbar spinal stenosis (lss), pain management

## Abstract

Lumbar spinal stenosis (LSS) occurs due to the narrowing of the space within the vertebral canal and or intervertebral foramina. This results in the compression of the spinal cord and possibly the roots of the spinal nerves. Lower back pain and neurogenic claudication (NC) are major symptoms of spinal stenosis. This is a literature review that summarizes the important findings pertaining to pain management of spinal stenosis. Twenty-four original articles were assessed. Pain can be treated through non-invasive or surgical methods. Conservative techniques include physical exercises, epidural corticosteroid injection, local anesthetic injection therapy, and oral analgesics. Surgical intervention deals with the decompression of the affected spinal region, with or without vertebral fusion surgery. Other novel surgical techniques include implantation of specific equipment, known as interspinous spacer devices and minimally invasive lumbar decompression (MILD). Most studies offering a comparative analysis have demonstrated that surgical intervention is more efficacious than non-surgical interventions to manage pain associated with spinal stenosis.

## Introduction and background

Lower back pain is one of the most prevalent complaints in outpatient orthopedic departments worldwide. The most common causes include lower vertebral fractures (often a result of trauma or degenerative disk disease), lower back sprain (usually due to heavy load-lifting or poor technique), intervertebral disc slip/sciatica, spinal tumors, and fibromyalgia [1]. Spinal stenosis occurs when there is a tight compression of the spinal cord or spinal nerves secondary to a narrowing of the vertebral canal space or intervertebral foramina space. There are many potential causes of spinal stenosis, including developmental abnormalities (e.g., achondroplasia) or age-related degenerative changes, such as osteoporosis, osteoarthritis, disc degeneration, and spondylosis. Narrowing of the vertebral canal or intervertebral foramina may be an implication of disc protrusion, osteophytic growth of the lumbar vertebrae, and the presence of short and thickened pedicles. Lumbar spinal stenosis (LSS) may also be secondary to genetic conditions, including ankylosing spondylitis, spondylosis, and spondylolisthesis [2].

The incidence of back pain is estimated to be between 7% and 37% depending on the population [3]. However, the mere presence of radiologic evidence of LSS does not necessarily correlate to having clinical symptoms. Kalichman et al. demonstrated that patients suffering from spinal stenosis have a three times higher risk of complaining of back pain than the population at large [4]. However, in addition to spinal pain, there are several other clinical manifestations associated with LSS. Spinal stenosis often presents with “neurogenic claudication” (NC), i.e., sharp and cramping pain in the lower limbs, exaggerated with walking and dependent on position. The positional dependent pain is often described as a “stabbing leg pain” that is brought on by extension of the spinal column (shopping-cart sign) or by standing, and the pain is often relieved with vertebral flexion. Cases involving a slipped disc may also present with recurrent episodes of sciatica [5]. 

LSS management includes conservative, non-invasive methods and invasive procedures. Prior to treatment, radiological analysis often confirms the clinical suspicion and demonstrates a narrowing of the spinal canal [5]. Initial treatments usually include pharmacologic analgesics, such as nonsteroidal anti-inflammatory drugs (NSAIDs) and opioids. Refractory cases may undergo continued epidural injection therapy via corticosteroids [6]. Besides pharmacological intervention, pain may be minimized with various exercises, including abdominal muscle exercises that reduce forward convexity of the lumbar column (lordosis). Interventions, such as treadmill walking and the use of spinal orthoses, have demonstrated efficacy in reducing pain [2].

In cases refractory to standard non-invasive therapy, surgery is the next step [7]. Surgery involves laminectomy of the involved vertebrae, resulting in spinal cord and nerve decompression. Subsequently, vertebral fusion surgery is often performed, providing additional mechanical support to the region weakened by surgery. However, there is limited data demonstrating further pain relief with fusion surgery coupled with the cord decompression technique compared to simple decompression. This study aims to summarize the key management strategies in the management of LSS, and both invasive and non-invasive modalities will be assessed.

## Review

Material and methods

This study is a literature review. All of the articles were obtained through PubMed Central (PMC) or Google Scholar. The following keywords were used: "spinal stenosis," "management of pain in spinal stenosis," and "surgical versus non-surgical management of spinal stenosis." A total of 41 articles were selected in the first phase. The selection criteria included clinical trials, retrospective studies, and case series that were relevant to the subject (from 1995 onwards). All systematic review and meta-analysis articles were excluded. After the exclusion, 24 articles were analyzed. These articles are listed in Tables 1-3.

**Table 1:** Summary of non-invasive management strategies

No.	Article title	Authors	Journal of publication	Year of publication	Main findings
1.	*Effects of Epidural Steroid Injection on Pain Due to Lumbar Spinal Stenosis or Herniated Disks: A Prospective Study *[8]	Rivest C, Katz JN, Ferrante FM, Jamison RN	Arthritis and Rheumatology	1998	This study involved 212 patients suffering from either spinal stenosis or disc herniation. All participants were treated with epidural steroid injections for symptomatic relief. Following the intervention, 38% of patients with lumbar stenosis demonstrated a statistically significant improvement in pain scores, whereas 61% of cases of herniated discs had a statistically significant improvement in pain status.
2.	*A Comparison Between Two Physical Therapy Treatment Programs for Patients With Lumbar Spinal Stenosis: A Randomized Clinical Trial *[9]	Whitman JM, Flynn TW, Childs JD, et al.	Spine	2006	Fifty-eight patients were divided into two groups. Patients of the first group underwent manual physiotherapy and treadmill walking. The other group performed lumbar exercises and treadmill walking. Follow-up sessions were done after six weeks and one year. The researchers concluded that manual physiotherapy, with treadmill walking, resulted in better outcomes when compared to lumbar exercises in terms of improvement in disability and other physiological functions.
3.	*Lumbar Interlaminar Epidural Injections in Central Spinal Stenosis: Preliminary Results of a Randomized, Double-Blind, Active Control Trial *[10]	Manchikanti L, Cash KA, McManus CD, Damron KS, Pampati V, Falco FJ	Pain Physician	2012	Approximately 120 patients with spinal stenosis were divided into two groups. One group received local anesthetic injection only and the other received local anesthetic + betamethasone. The Oswestry Disability Index was used as a data collection tool. Subsequent data analysis demonstrated that both groups had a marked improvement in pain intensity: The first group had a 70% improvement rate, while the second group experienced a 63% improvement rate.
4.	*Fluoroscopic Caudal Epidural Injections With or Without Steroids in Managing Pain of Lumbar Spinal Stenosis One-Year Results of Randomized, Double-Blind, Active-Controlled Trial* [11]	Manchikanti L, Cash KA, McManus CD, Pampati V, Fellows B	Journal of Spinal Disorders and Techniques	2012	In this randomized trial, 100 patients were divided into two groups. Group 1 was treated with an epidural injection of 0.5% lidocaine, while patients in group 2 received 9 ml of 0.5% lidocaine mixed with 1 ml of a corticosteroid. Almost 48% of group 1 members and 46% of group 2 members demonstrated statistically significant improvement in pain intensity.
5.	*Results of 2-Year Follow-Up of a Randomized, Double-Blind, Controlled Trial of Fluoroscopic Caudal Epidural Injections In Central Spinal Stenosis *[12]	Manchikanti L, Cash KA, McManus CD, Pampati V, Fellows B	Pain Physician	2012	This randomized double-blinded study divided 100 patients with spinal stenosis into two groups that both received spinal epidural injections. The first group only received local anesthetic, while the second group received both local anesthetic and corticosteroid. Improvement of pain and functionality was observed in both groups (38% vs. 44%, respectively).
6.	*A Randomized, Double-blind Controlled Trial of Lumbar Interlaminar Epidural Injections in Central Spinal Stenosis: 2-year Follow-up* [13]	Manchikanti L, Cash KA, McManus CD, Damron KS, Pampati V, Falco FJ	International Physical Medicine and Rehabilitation Journal	2014	This randomized trial divided 120 patients into two groups. Group 1 was treated with lumbar epidural lidocaine (0.5%) 6 ml, and group 2 received 1 ml steroid preparation mixed with 5 ml lidocaine. The Numerical Pain Rating Scale and Oswestry Disability Index were used to assess patient status. At two years' follow-up, both groups demonstrated no significant difference with respect to improvement rates (group 1 had 72% and group 2 had 73% pain relief).
7.	*Pregabalin for Refractory Radicular Leg Pain due to Lumbar Spinal Stenosis: A Preliminary Prospective Study* [14]	Orita S, Yamashita M, Eguchi Y, et al.	Pain Research and Management	2016	Researchers tested the efficacy of pregabalin in pain management in 104 cases of lumbar stenosis. Every patient in this study demonstrated symptoms of neurogenic claudication refractory to nonsteroidal anti-inflammatory drugs (NSAIDs). Statistically significant improvement in terms of pain control and gait disturbance was observed; however, dizziness was an associated complication of therapeutic intervention.

**Table 2:** Summary of invasive management techniques

No.	Article title	Authors	Journal of publication	Year of publication	Main findings
1.	*Surgery of the Lumbar Spine for Spinal Stenosis in 118 Patients 70 Years of Age or Older* [15]	Ragab AA, Fye MA	Spine	2003	This retrospective study collected data from 118 subjects who had undergone surgical treatment for lumbar spinal stenosis. Patients were assessed for post-surgical morbidity and their satisfaction with surgery. Overall morbidity was approximately 20%, while 109 patients were satisfied with their surgical outcome and were able to continue their daily life routine post-operatively.
2.	*Predominant Leg Pain is Associated With Better Surgical Outcomes in Degenerative Spondylolisthesis and Spinal Stenosis: Results From the Spine Patient Outcomes Research Trial (Sport)* [16]	Pearson A, Blood E, Lurie J, Abdu W, Sengupta D, Frymoyer JW, Weinstein J	Spine	2011	In this study, two patient groups were organized: the degenerative spondylolisthesis group (591) and spinal stenosis group (615). About 62% of cases from each group underwent surgical intervention. The patients in either cohort were further classified into three categories with respect to their symptoms: leg pain predominant, lower back pain predominant, and mixed symptoms. The researchers subsequently demonstrated that leg pain symptoms were more likely relieved by surgical intervention.
3.	*Functional and Patient‐Reported Outcomes in Symptomatic Lumbar Spinal Stenosis Following Percutaneous Decompression *[17]	Mekhail N, Costandi S, Abraham B, Samuel SW	Pain Practice	2012	Forty patients were recruited for percutaneous lumbar spinal decompression. The Pain Disability Index and Roland-Morris Questionnaire were used to assess the patients’ status. Both tools demonstrated significant progress with respect to the overall functional profile and a decrease in pain intensity and disability.
4.	*A Randomized, Controlled Trial of Fusion Surgery for Lumbar Spinal Stenosis* [18]	Försth P, Ólafsson G, Carlsson T, et al.	The New England Journal of Medicine	2016	A total of 247 patients with spinal stenosis aged between 50 and 80 years were randomly categorized to undergo either of the following two operations; spinal decompression with fusion surgery or decompression only. The data collection tool used was the Oswestry Disability Index. The findings demonstrated no statistically significant difference between the two groups with regard to patient recovery rates. However, hospital-stay duration of fusion surgery individuals was almost twice that of those who underwent decompression only. Moreover, bleeding complications were more likely to be seen in the former group.
5.	*Long-Term Safety and Efficacy of Minimally Invasive Lumbar Decompression Procedure for the Treatment of Lumbar Spinal Stenosis With Neurogenic Claudication 2-Year Results of MiDAS ENCORE* [19]	Staats PS, Chafin TB, Golovac S, et al.	Regional Anesthesia and Pain Medicine	2018	In this trial, two groups of patients were formed: the first (143) were treated with MILD surgery (minimally invasive lumbar decompression), while the second (131) were treated with epidural steroid injections. The Oswestry Disability Index and other scores showed high improvement rates for surgically treated cases. Only 1.3% of the patients experienced surgical complications.
6.	*Clinical Outcome After Surgery for Lumbar Spinal Stenosis in Patients With Insignificant Lower Extremity Pain. A Prospective Cohort Study From the Norwegian Registry for Spine Surgery *[20]	Hermansen E, Myklebust TÅ, Austevoll IM, et al.	BMC Musculoskeletal Disorders	2019	In this study, data obtained from 3,181 patients who underwent spine decompression surgery were assessed. They divided patients into four groups with respect to pain status: Group 1 with 154 cases, group 2 with 753 cases, group 3 with 1,766 patients, and group 4 with 528 patients. The pain status of the four groups varied from group 1 to 4 as insignificant, mild to moderate, severe, and extremely severe pain. The Oswestry Disability Index was used to monitor post-surgical outcomes. At 12 months' follow-up, the group 1 members reported minimum improvement from pre-surgical evaluation as compared to all the other groups.
7.	*Minimally Invasive Treatment of Lumbar Spinal Stenosis With a Novel Interspinous Spacer* [21]	Shabat S, Miller LE, Block JE, Gepstein R	Clinical Interventional Aging	2011	In this study, 53 patients with LSS (lumbar spinal stenosis) were treated with the Superior (®) Interspinous Spacer (Vertiflex Inc.) and follow-up visits at five weeks, one year, and two years. The study endpoints utilized axial and extremity pain severity with an 11-point numeric rating scale, Zurich Claudication Questionnaire (ZCQ), and back function with the Oswestry Disability Index. Axial and extremity pain decreased by 54% over the two-year follow-up period. ZCQ symptom severity scores improved by 43%, and ZCQ function improved by 44% from pre-treatment to two years post-treatment. Moderate LSS can be effectively treated with a minimally invasive interspinous spacer. The device is appropriate for certain patients who have failed nonoperative treatment measures for LSS and strict anatomical criteria.
8.	Minimally Invasive Lumbar Decompression: A Review of Indications, Techniques, Efficacy, and Safety [22]	Jain S, Deer T, Sayed D, et al.	*Pain Management*	2020	This study utilized an extensive literature review of two randomized controlled trials, together with 11 other controlled clinical studies, to establish the efficacy of MILD surgery (minimally invasive lumbar decompression). This study recommended that MILD should be considered as the first intervention after failure of conservative measures for patients diagnosed with lumbar spinal stenosis (LSS) and ligamentum flavum hypertrophy (LFH) ≥2.5 mm.

**Table 3:** Summary of studies that performed a comparative analysis of surgical versus non-surgical management

No.	Article title	Authors	Journal of publication	Year of publication	Main findings
1.	*The Maine Lumbar Spine Study, Part III: 1-Year Outcomes of Surgical and Nonsurgical Management of Lumbar Spinal Stenosis* [23]	Atlas SJ, Deyo RA, Keller RB, Chapin AM, Patrick DL, Long JM, Singer DE	Spine	1996	This trial involved 148 patients with spinal stenosis: 81 were surgically treated, while the remaining were given conservative therapy. Most patients undergoing surgery had severely intense pain and other symptoms, while the majority of patients treated conservatively experienced mild to moderate symptoms. At one-year follow-up, 55% of the patients treated surgically demonstrated improvement when compared to the 28% managed conservatively. This difference remained statistically significant even after adjustments were made for initial symptom severity.
2.	*Surgical and Nonsurgical Management of Lumbar Spinal Stenosis: Four-Year Outcomes From the Maine Lumbar Spine Study* [24]	Atlas SJ, Keller RB, Robson D, Deyo RA, Singer DE	Spine	2000	Out of a total of 148 patients with lumbar spinal stenosis, 119 patients were compared for their prognosis after a period of four years. Sixty-seven were surgically intervened, and 52 were treated non-invasively. After a four-year follow-up, the patients that underwent surgical intervention demonstrated significantly improved pain status (70% > 52%). More than 60% of the surgical patients were satisfied with their current health status, while ~40% of patients treated non-surgically felt the same.
3.	A Prospective Randomized Multi-center Study for the Treatment of Lumbar Spinal Stenosis With the X STOP Interspinous Implant: 1-year Results [25]	Zucherman JF, Hsu KY, Hartjen CA, et al.	European Spine Journal	2004	In this study, researchers used an interspinous spacer device (X STOP implant) for 100 spinal stenosis patients, whereas 91 subjects were treated conservatively. The Zurich Claudication Questionnaire (ZCQ) was used as a data collection and analysis tool. X STOP implant therapy showed a high improvement rate of >50%, while the control group showed a 10% success at six weeks post-treatment. Similar results were obtained at six and 12 months' follow-up.
4.	Surgical or Nonoperative Treatment for Lumbar Spinal Stenosis?: A Randomized Controlled Trial [26]	Malmivaara A, Slätis P, Heliövaara M, et al.	Spine	2007	This randomized controlled trial consisted of 94 patients suffering from lumbar spinal stenosis: 53.2% (50 patients) underwent laminectomy and fusion surgery. The remaining 44 were treated conservatively. The Oswestry Disability Index was utilized to compare the post-treatment status of patients. In subsequent follow-up sessions, the patients treated surgically demonstrated decreased disability, lower limb pain, and back pain. However, the walking ability improved equally in both groups.
5.	*The Preliminary Results of a Comparative Effectiveness Evaluation of Adhesiolysis and Caudal Epidural Injections in Managing Chronic Low Back Pain Secondary to Spinal Stenosis: A Randomized, Equivalence Controlled Trial* [27]	Manchikanti L, Cash KA, McManus CD, Pampati V, Singh V, Benyamin R	Pain Physician	2009	The authors divided patients into two groups with 25 patients each (total = 50). One group was given epidural injections with a mixture of local anesthetic, isotonic NaCl (0.9%) solution, and betamethasone. The other group received hypertonic (10%) saline, betamethasone, and lidocaine injection. This experimental group also underwent adhesiolysis. Follow-up analysis resulted in pain control in approximately 76% cases of the intervention group compared to 4% of the control group population.
6.	*Surgical versus Non-Operative Treatment for Lumbar Spinal Stenosis Four-Year Results of the Spine Patient Outcomes Research Trial (SPORT)*[28]	Weinstein JN, Tosteson TD, Lurie JD, et al.	Spine	2010	The study performed a comparison of surgical laminectomy versus non-invasive treatment strategies. A total of 289 patients were included in the randomized group, while 365 patients were recruited to the observational cohort. The results of the study showed improved function in surgically treated patients, even at four years postoperatively.
7.	*A Double‐blind, Randomized, Prospective Study of Epidural Steroid Injection vs. The MILD ® Procedure in Patients with Symptomatic Lumbar Spinal Stenosis *[29]	Brown LL	Pain Practice	2012	In this study, 38 patients underwent intervention for lumbar spinal stenosis. Twenty-one were surgically treated for spinal decompression (MILD procedure), while the other 17 underwent epidural steroid injection therapy. At follow-up, surgically intervened patients demonstrated greater improvement with respect to overall functional status and a decrease in pain intensity.
8.	Effects of Transforaminal Balloon Treatment in Patients With Lumbar Foraminal Stenosis: A Randomized, Controlled, Double-blind Trial [30]	Kim SH, Choi WJ, Suh JH, et al.	Pain Physician	2013	A total of 62 patients were randomized and treated with either steroid injections with transforaminal balloon intervention or with transforaminal injections alone. The first group demonstrated better post-intervention results when compared to the latter. Nearly 20% of cases in the balloon therapy group maintained their pain-free status after one year, as compared to 0% in the other group.
9.	*Surgery Versus Nonsurgical Treatment of Lumbar Spinal Stenosis* [31]	Delitto A, Piva SR, Moore CG, et al.	Annals of Internal Medicine	2015	In this project, 169 patients with spinal stenosis patients were split into two groups: 87 were treated surgically and 82 were treated with physiotherapeutic interventions. Upon follow-up analysis, patient functional status was improved in a total of 22.4% of patients who underwent surgery, compared to 19.2% patients who underwent physiotherapy. No statistically significant differences were seen between the two groups.

This literature review summarizes some of the most important findings from several original articles. The foundational pain management strategies for spinal stenosis include non-invasive measures, such as physiotherapy, epidural steroid injections (ESIs), NSAIDs, and pregabalin. The key surgical measures include lumbar spinal decompression, with or without fusion surgery, intraspinal implants, and minimally invasive lumbar decompression (MILD). 

ESIs are non-invasive procedures that are performed to relieve lower back pain. During the procedure, a physician injects a combination of a steroid and a local anesthetic into the epidural space around the spinal cord and nerves in the lumbar region. The epidural space is the space between the outermost layer of the spinal cord and the vertebral canal that contains fat, nerve roots, blood vessels, and lymphatics [8]. There are three approaches that are used: the transforaminal, interlaminar, and caudal approaches. The injection of steroid and local anesthetic into the epidural space reduces inflammation, which can help reduce neuropathic pain symptoms, such as numbness and tingling. Similar with many procedures, patients can vary in their response, with some individuals experiencing significant pain relief, while others not receiving much of a benefit. Epidural injections can provide temporary relief; however, they are not a long-term solution and can only be repeated three to four times a year [8]. They also have long-term side effects from the steroid component of the injection, such as hyperglycemia, infection, bleeding, and osteoporosis [10].

A laminectomy is a surgical procedure that is performed to treat LSS. During a laminectomy, the surgeon removes a portion of the vertebra called the lamina [6]. This helps to reduce pressure on the spinal cord and nerves by providing more space within the canal. The process involves first making an incision over the lumbar spine. Following the incision, soft tissue dissection occurs in which the muscles and tissues are moved aside carefully to expose the lamina. The surgeon then removes a section of the lamina, which creates more space in the spinal canal. Other elements, such as hypertrophic ligaments or osteophytes, may be removed as well if they are contributing to the compression of the spinal cord or nerve roots [6]. If the spine exhibits signs of instability, a fusion may be required in those affected vertebrae.

After the surgery, patients are observed for a short time before being discharged. Patients will usually start physical therapy immediately following surgery. Some patients will go to inpatient rehabilitation facilities as they may be extremely debilitated following surgery and have multiple complex conditions that will benefit from a stay in an inpatient facility. Laminectomies are invasive procedures that carry risks, such as paralysis, infection, bleeding, and post-laminectomy syndrome [15]. The typical recovery rate can vary from patient, but most patients typically take a year to recover after the surgery [15].

Kovacs et al. [6] performed a systematic review of treatment of spinal stenosis; they analyzed data from over 900 patients. Prior to surgical treatment, conservative options were adopted, including orthoses, physiotherapy, and other walking exercises. Moreover, several subjects underwent medical treatment with NSAIDs or ESIs. If and when pharmacologic therapies failed, the patients underwent surgical spinal decompression. This resulted in a remarkably efficient resolution of symptoms, including pain, and other pre-surgical complaints, such as difficulty in walking. 

In regard to non-invasive management, the most common therapeutic regimen used for relieving pain is usually epidural corticosteroid injection therapy (possibly accompanied with a local anesthetic). In a study reviewing 13 randomized controlled trials (RCTs), "steroid plus local anesthetic" and "local anesthetic alone" regimens yielded potentially similar therapeutic effects. Pain and other symptoms gradually improved for both treatment groups [32]. Apart from pharmacological intervention, physiotherapy exercises improved pain status and gait disturbances. Several researchers found it effective when compared to the steroid injection regimen [33]. 

Lumbar spinal decompression surgery has been characterized as the “game-changing” treatment for spinal stenosis. Notable parameters used to predict surgical outcome include but are not limited to preoperative depression, presence of comorbidities, and spinal stenosis due to scoliosis [34]. Machado et al. demonstrated that post-surgical patient prognosis remains nearly the same for patients undergoing combined decompression plus fusion surgery compared to those treated with decompression alone [35]. Interspinous spacing devices carry a higher success rate than decompression surgery; however, they are more likely associated with an increased risk for re-surgery [36]. 

The usage of the Vertiflex interspinous spacer is a minimally invasive medical device designed to treat LSS when patients show signs of symptomatic NC and have failed conservative treatment. Moreover, the majority of patients dealing with spinal stenosis are elderly with multiple comorbidities. Shabat et al. showcased 53 patients (mean age of 70 ± 11 years) with intermittent NC to moderate LSS, who presented a decrease in axial and extremity pain by 54% over a two-year period with no device infection, implant breakage, and migration [21]. An alternative treatment to LSS is MILD due to the primary reason of reducing trauma around the surrounding tissue, allowing for smaller incisions, resulting in minimal damage to muscles and ligaments surrounding the back. Two RCTs together with 11 other controlled studies have established the efficacy of mild demonstrating superior safety profile equivalents to ESIs [22]. Moreover, MILD is recommended as the first intervention after failure of conservative measures for LSS with symptoms of NC and ligamentum flavum hypertrophy [29]. However, each treatment depends on the individual patient's symptoms and current medical history. If a patient exhibits signs of ligamentum flavum hypertrophy on MRI and determined to be the pain generator, then the MILD procedure would be preferred to reduce the patient's pain. The MILD procedure is also indicated for stenosis at the L5-S1 level, while the Vertiflex implant cannot be used at the L5-S1 level [29]. The Vertiflex procedure can address more causes of spinal stenosis, such as facet hypertrophy, and stenosis due to disc herniation.

## Conclusions

This literature review examined the current pain management interventions for LSS and detailed the various strategies to help patients. Physical therapy, medications, and procedures, such as epidural injections, are non-invasive interventions that can alleviate symptoms and have consistent evidence to support their use.

Patients with severe symptoms and/or neurological deficits will often require surgeries, such as laminectomies. However, proceeding with surgery requires a careful discussion between the patient and the surgeon regarding the potential risks and benefits. There are newer devices and procedures, such as intervertebral spacer devices, and procedures, such as MILD, that may allow patients to avoid the risks of surgery and experience symptom relief.

In the end, providing the best outcome to the patient entails a multidisciplinary approach among pain specialists, surgeons, physical therapists, and patients themselves. The decision to select a treatment requires a careful consideration of a patient’s goals, preferences, and tolerability to the risks associated with each intervention. As researchers continue to develop new techniques for managing LSS, healthcare professionals will be better suited to treat their patients.
